# Linking biodiversity and ecosystems: towards a unifying ecological theory

**DOI:** 10.1098/rstb.2009.0155

**Published:** 2010-01-12

**Authors:** Michel Loreau

**Affiliations:** Department of Biology, McGill University, 1205 Avenue Docteur Penfield, Montreal, Quebec H3A 1B1, Canada

**Keywords:** biodiversity, community, ecosystem, ecology, evolution, theory

## Abstract

Community ecology and ecosystem ecology provide two perspectives on complex ecological systems that have largely complementary strengths and weaknesses. Merging the two perspectives is necessary both to ensure continued scientific progress and to provide society with the scientific means to face growing environmental challenges. Recent research on biodiversity and ecosystem functioning has contributed to this goal in several ways. By addressing a new question of high relevance for both science and society, by challenging existing paradigms, by tightly linking theory and experiments, by building scientific consensus beyond differences in opinion, by integrating fragmented disciplines and research fields, by connecting itself to other disciplines and management issues, it has helped transform ecology not only in content, but also in form. Creating a genuine evolutionary ecosystem ecology that links the evolution of species traits at the individual level, the dynamics of species interactions, and the overall functioning of ecosystems would give new impetus to this much-needed process of unification across ecological disciplines. Recent community evolution models are a promising step in that direction.

## 1. Strengths and weaknesses of community ecology and ecosystem ecology

One of the distinctive and fascinating features of ecological systems is their extraordinary complexity. An ecosystem is often composed of thousands of different species that interact in myriad different ways at the scale of a single hectare. These complex local systems are strongly connected to each other and aggregate into larger and larger entities from the landscape scale to that of the entire biosphere, where it becomes evident that they exert a major influence on the physical and chemical properties of our planet. How can such enormously complex systems be studied?

During the second half of the twentieth century, two increasingly divergent approaches to ecological systems developed within ecology, which have gradually led to two distinct disciplines, community ecology and ecosystem ecology. A community is defined broadly as a set of species that live together in some place. The focus in community ecology has traditionally been on species diversity: what exogenous and endogenous forces lead to more or less diverse communities? How do species interactions constrain the number of species that can coexist? What patterns emerge from these species interactions? An ecosystem is the entire system of biotic and abiotic components that interact in some place. The ecosystem concept is broader than the community concept because it includes a wide range of biological, physical, and chemical processes that connect organisms and their environment. But the focus on ecosystem ecology has traditionally been on the overall functioning of ecosystems as distinct entities: how is energy captured, transferred, and ultimately dissipated in different ecosystems? How are limiting nutrients recycled, thereby ensuring the renewal of the material elements necessary for growth? What factors and processes control energy and material flows, from local to global scales?

In a sense, community ecology provides a microscopic perspective on ecosystems because it analyses their parts, while ecosystem ecology provides a macroscopic perspective on the same systems because it studies them as a whole. The distinction between micro- and macroscopic, however, does not necessarily apply to the spatial scales considered by the two disciplines. Although much of community ecology does consider species interactions at small scales, a growing fringe, known as macroecology, considers patterns of species diversity and species distributions at vast spatial scales. The focus on species—species distributions, species diversity, species interactions—is more central to the community ecological approach than the spatial scale considered. Similarly, ecosystem ecology studies the fluxes of energy and materials at various spatial scales. What distinguishes the ecosystem ecological approach is its focus on the system as a whole, irrespective of the species that compose it.

At a time when humankind rises to the status of a major global biogeochemical force and raises the prospect of a global ecological crisis, it seems appropriate to step back and ask whether individually studying communities and ecosystems is the best path to follow. Human environmental impacts include the destruction and fragmentation of natural habitats, pollution, climate change, overexploitation of biological resources, homogenization of biota and biodiversity loss. These impacts affect species and ecosystems indistinctly. Moreover, they interact with each other, which may lead to non-additive cumulative effects. For instance, climate change is likely to cause massive additional biodiversity loss. Biodiversity loss in turn is likely to decrease the ability of ecosystems to resist the effects of climate change, with possible feedbacks on the climate system itself. Species, communities and ecosystems have always been inextricably linked, but the major disruptions generated by humans in the current period make this reality plainly obvious. A synthetic approach to ecology, which integrates populations, communities and ecosystems, is required to develop appropriate responses to the global ecological crisis we are entering.

Lawton (1999) recently drew a rather dark picture of the current status of community ecology, ‘where there are a large number of case histories, and very little other than weak, fuzzy generalisations’. The community is indeed the hierarchical level where the basic characteristics of life—its diversity, complexity and historical nature—are perhaps most daunting and challenging. Community ecology is a dynamic field of research in which knowledge has accumulated rapidly during the past 50 years or so based largely on a modern hypothetico-deductive approach. But it is replete with poorly tested theories and hypotheses, which hinders steady scientific progress. As a result, it has few laws or robust generalizations, except for some large-scale empirical patterns such as species–area relationships (Rosenzweig 1999). Despite the proliferation of theories and hypotheses, however, a large amount of robust results have been obtained on a wide range of small-scale ecological processes. What is lacking is a synthetic theoretical framework to organize these results into a coherent set of alternative or complementary hypotheses that yield testable predictions. For instance, there are many theories of species coexistence, each of which invokes a different mechanism. But these various mechanisms can be organized into a limited number of classes based on the way these mechanisms allow competitive exclusion to be avoided, as in Chesson's (2000) distinction between equalizing and stabilizing mechanisms. If distinctions such as this were made operational through the development of appropriate quantitative measures and if their implications for the functioning of entire communities or ecosystems were clearly identified, they would be more readily amenable to empirical tests, they would foster unification of studies of species coexistence and they would yield valuable robust insights that could be used in broader and more applied contexts.

In contrast, ecosystem ecology is an ecological discipline that has traditionally had a strong empirical basis. Its theories are largely based on inductive generalizations from field measurements, with comparatively few theory-driven hypotheses and experimental tests. There is no doubt that the ecosystem approach has been instrumental in developing our understanding of the global biogeochemistry of the Earth system and of current global environmental changes. Yet, despite these successes, a number of authors have questioned its static view of ecological systems and even its scientific relevance, calling for a fundamental rejuvenation of the discipline (O'Neill 2001). Two key elements that are still insufficiently developed in ecosystem ecology are a strong hypothetico-deductive approach and proper consideration of the internal dynamics and complexity of ecological systems. Strengthening theory, experimental tests and their interaction, and paying due attention to ecological dynamics and complexity would probably contribute to make ecosystem ecology a more dynamic and attractive scientific discipline.

On balance, then, it appears that community ecology and ecosystem ecology provide two perspectives on complex ecological systems that have largely complementary strengths and weaknesses. Both disciplines have been called into question, and each would benefit from the perspective developed by the other. Developing theories about interactions between species and between these and their environment, with the ultimate goal of predicting ecosystem functioning and ecosystem services, would help to focus community ecology on issues that are both scientifically important and socially relevant. Incorporating the diversity, complexity and dynamical nature of communities in its view of ecosystem functioning would help ecosystem ecology to be livelier and to provide more reliable, if probably more uncertain, predictions. It is becoming increasingly clear that merging the two perspectives is necessary both to ensure continued scientific progress and to provide society with the scientific means to face growing environmental challenges.

The need for integration of community ecology and ecosystem ecology has been increasingly recognized during the past 20 years. There have been a number of attempts at doing so from a variety of perspectives, such as those provided by hierarchy theory (O'Neill *et al*. 1986), linking nutrient cycling and food webs (DeAngelis 1992), linking species and ecosystems (Jones & Lawton 1995), complex systems theory (Levin 1999; Solé & Bascompte 2006), ecological stoichiometry across levels of biological organization (Sterner & Elser 2002) and the metabolic theory of ecology (Brown *et al*. 2004). Each of these perspectives has contributed to addressing part of the problem. But a broader synthesis is still needed (Loreau in press). Here I will show how the new biodiversity and ecosystem functioning research field has contributed to this goal, before turning to what I see as a major scientific challenge for the future.

## 2. Biodiversity and ecosystem functioning: an integrative approach

The relationship between biodiversity and ecosystem functioning has emerged as a central issue in ecological and environmental sciences during the past 15 years. The idea that greater plant diversity allows greater plant biomass production dates back to Darwin (McNaughton 1993; Hector & Hooper 2002), but it was only in the 1990s that the interest in the effects of biodiversity on ecosystem functioning penetrated experimental and theoretical ecology. This interest spread very rapidly, leading to an entire new research field at the interface between community ecology and ecosystem ecology (Schulze & Mooney 1993; Tilman 1999; Chapin *et al*. 2000; Loreau 2000; Kinzig *et al*. 2001; Loreau *et al*. 2001; Loreau *et al*. 2002*b*; Hooper *et al*. 2005; Balvanera *et al*. 2006; Cardinale *et al*. 2006; Cardinale *et al*. 2007).

Interest in this issue grew largely out of practical concerns about the potential ecological consequences of current biodiversity loss caused by the increased impact of human activities on natural and managed ecosystems. There is growing recognition that the world's ecosystems provide society with a wide range of ecological ‘services’ that are crucial to human well-being and sustainable development (Millennium Ecosystem Assessment 2005). These services are derived from the normal functioning of ecosystems, raising the important question of whether impoverished ecosystems may in some way function less efficiently than the more species-rich systems from which they are derived, and hence gradually lose their ability to deliver ecosystem services to human societies. But beyond this eminently practical motivation, the new biodiversity and ecosystem functioning research field has had a much broader and deeper transformative role in ecology.

### (a) Fostering integration of community ecology and ecosystem ecology

One of its main benefits has been to foster integration of community ecology and ecosystem ecology. Ecology has traditionally regarded, implicitly or explicitly, species diversity as an epiphenomenon driven by a combination of abiotic environmental factors (such as temperature, rainfall and soil fertility), ecosystem processes that are themselves determined by these abiotic factors (such as productivity, biomass and nutrient cycling) and biotic interactions within communities (such as competition and predation). This tenet is shared by community ecology and ecosystem ecology. Community ecology has been devoted historically to explaining patterns and processes of species coexistence and diversity. Ecosystem ecology has often ignored species diversity as some sort of ‘background noise’ irrelevant to ecosystem functioning. Thus, the two disciplines have considered species diversity in contrasting ways, which explains their historical divergence. But both have shared the basic assumption that species diversity is an epiphenomenon. The new biodiversity and ecosystem functioning research field has overthrown this central tenet by considering biodiversity—in particular species and genetic diversity—as a driver of ecosystem functioning (Naeem 2002).

**Figure 1.**
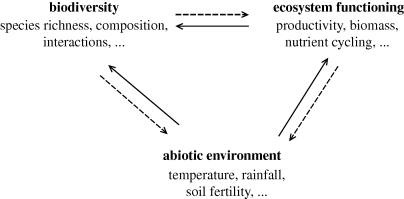
Ecology has traditionally regarded biodiversity as an epiphenomenon driven by the abiotic environment and ecosystem functioning (solid arrows). Recent biodiversity and ecosystem functioning research has focused on the reverse effect of biodiversity on ecosystem functioning (thick dashed arrow). The ecosystem engineering and niche construction concepts further seek to capture biological feedbacks on the abiotic environment (thin dashed arrows).

In a way, this change may be viewed as a simple extension of the classical paradigm, in which an additional arrow pointing from biodiversity to ecosystem functioning completes the picture of causalities between abiotic factors, ecosystem functioning and biodiversity (figure 1). This apparently simple addition, however, has far deeper consequences than appears at first sight. The presumed lack of functional consequences of biodiversity is implicitly what justified the separation of community ecology and ecosystem ecology: ecosystem ecologists could study ecosystems as though these were independent of biodiversity and the community processes that maintain it, while community ecologists could study biodiversity and the processes that affect it without explicitly considering the ecosystems in which communities are embedded, by treating ecosystem processes as external constraints. If biodiversity affects ecosystem functioning, ecosystem ecologists can no longer ignore the dynamics of biodiversity within ecosystems. Similarly, community ecologists can no longer ignore the potential feedback that biodiversity has on its own maintenance through ecosystem functioning. The basis for the historical separation of the two disciplines then vanishes, even though it may take some time until both sides recognize the full implications of this change.

### (b) The controversy over experimental results

**Figure 2.**
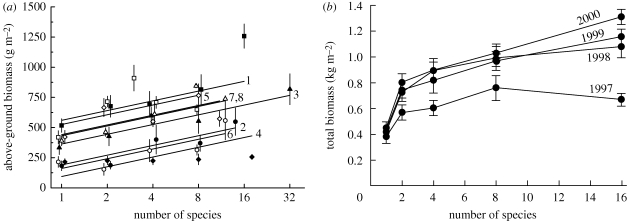
Effects of plant species richness (*a*) on annual above-ground plant biomass production in the BIODEPTH experiment and (*b*) on annual total plant biomass production in the Cedar Creek experiment. Average biomass production increased with plant diversity across the eight sites in the BIODEPTH experiment, an effect that became stronger through time in the Cedar Creek experiment. In (*a*) lines are regression slopes and symbols (staggered for clarity) are richness level means and standard errors: filled squares = Germany, line 1; filled circles = Portugal, line 2; filled triangles = Switzerland, line 3; filled diamonds = Greece, line 4; open squares = Ireland, line 5; open circles = Sweden, line 6; open diamonds = Sheffield (UK), line 7 and Silwood Park (UK), line 8. Modified from Hector *et al*. (2002) and Tilman *et al*. (2001).

Recent work has now clearly established that biodiversity does indeed affect ecosystem processes. The strongest evidence to date comes from field experimental studies that have manipulated plant species richness in temperate grasslands. Two of the largest such experiments have been the Cedar Creek biodiversity experiment in Minnesota, USA (Tilman *et al*. 1997; Tilman *et al*. 2001) and the BIODEPTH experiment in Europe (Hector *et al*. 1999; Spehn *et al*. 2005). The advantage of BIODEPTH is that it was replicated over eight sites under different biogeographic, climatic and soil conditions across Europe, which allowed testing the generality of biodiversity effects. The advantage of the Cedar Creek experiment is that it was run for more than a decade, which allowed testing the robustness of biodiversity effects through time. The two experiments provided very similar results overall. BIODEPTH showed a log-linear increase in plant above-ground biomass production with species richness across sites (figure 2*a*). The Cedar Creek experiment showed a positive response of total plant biomass production to species richness, which became stronger through time (figure 2*b*). The number of plant functional groups also had positive effects on plant biomass production in both cases.

These experimental results, however, generated a vigorous debate within the scientific community. Although they were consistent with expectations from niche theory, other theories suggested that overyielding (i.e. the fact that mixtures of several species yield more than expected based on the yield of these species in monoculture) and niche differentiation might not be the rule in plant communities. This prompted other hypotheses about the mechanisms that could generate the effects observed in the experiments. In particular, the ‘sampling effect’ hypothesis proposes that mean biomass production increases with plant species richness in experiments simply because of the increased probability of including a highly productive species in high-diversity plots (Huston 1997). Though sometimes heated, this debate ultimately revealed two of the main strengths of the new biodiversity and ecosystem functioning research field, i.e. its ability to link theory and experiments, and its ability to reach consensus despite strongly diverging initial views.

### (c) Linking theory and experiments

Ecology has had an unfortunate propensity to disconnect empirical and theoretical research, with a profusion of poorly generalized empirical data and an equal profusion of poorly tested theories. As a result of this disconnection, there have been few attempts at resolving controversies through consensus building within the ecological scientific community, leading to periodic shifts in fashionable research topics. In this context, the scientific process through which the biodiversity and ecosystem functioning research field developed is quite remarkable. At first, experiments outpaced theory, but soon theory played a key role in the resolution of the controversy raised by experimental results.

Scientific controversies are often the result of a lack of clarity in the theoretical framework, a lack of appropriate tools or a lack of sufficient empirical evidence to distinguish among clearly identified competing hypotheses. This one was no exception and combined all three problems. Therefore, the first step in its resolution was a theoretical advance in the conceptual framework in which the experiments were being conceived and interpreted. New theoretical work identified two main classes of mechanisms by which biodiversity influences productivity or other ecosystem processes, leading to two types of biodiversity effects: (i) functional complementarity—the complementarity effect, and (ii) selection of particular functional traits that affect species' competitive abilities—the selection effect (Loreau 2000). This apparently straightforward clarification was instrumental in resolving the debate over biodiversity experiments in several ways. First, it became clear that biodiversity matters only for ecosystem functioning to the extent that it provides phenotypic trait variation, or functional diversity, related to the particular ecosystem process considered. Second, it became also clear that the sampling effect is but a special, extreme case of the more general selection effect. Since selection and complementarity are two processes that operate concomitantly in competitive systems, this clarification contributed to build a consensus conceptual framework on biodiversity and ecosystem functioning (Loreau *et al*. 2001). Third, the analogy between the ecological selection effect and the evolutionary process of natural selection helped appreciate the selection effect as a relevant biological effect, not a mere statistical artefact.

After clarification of the theoretical framework in which biodiversity experiments can be properly interpreted, the second step in the resolution of the controversy consisted in devising new theoretical tools to analyse their results. Since several experiments were already completed at a considerable cost, how could the data collected in these experiments be used to assess the respective contributions of the selection and complementarity effects? A new additive partition methodology inspired by the Price equation in evolutionary genetics provided a powerful tool to separate the two effects (Loreau & Hector 2001). A large number of experimental studies have now used this additive partition. These have showed that selection and complementarity often co-occur in biodiversity experiments, but that positive net biodiversity effects are predominantly driven by functional complementarity between species, and further that this complementarity tends to increase through time (Cardinale *et al*. 2007).

The tight link between theory and experiments is what made the resolution of the debate possible scientifically. Experiments helped to build a more practically oriented theory, and theory helped to analyse and interpret experiments more effectively. This interactive process allowed clear but balanced conclusions to be drawn, thereby providing healthy ground for future studies.

### (d) Building scientific consensus

Resolving a controversy also has a human dimension—as does the scientific process in general. Bringing scientists with different views together with the explicit goal to move beyond existing divergences and build consensus without compromising on scientific rigour played an important part in the progress achieved in the biodiversity and ecosystem functioning research field. When I, Shahid Naeem and Pablo Inchausti convened the Conference ‘Biodiversity and ecosystem functioning: Synthesis and perspectives’ held in Paris in December 2000, the debate was becoming very acrimonious as a result of growing misunderstandings. At the end of the meeting, this acrimony had largely dissipated, common scientific grounds on a few key issues could be found, and scientific evidence based on rigorous theory and experimental data prevailed again in the subsequent scientific process. Two influential consensus papers came out of this meeting (Loreau *et al*. 2001; Hooper *et al*. 2005). These papers articulate a general framework for biodiversity and ecosystem functioning research, and provide a detailed assessment of scientific certainties and uncertainties in this area. I am convinced that efforts to build consensus and assess current knowledge and future challenges would help make ecology a more scientifically dynamic and socially relevant science. An international mechanism of scientific expertise on biodiversity and ecosystem services could provide a global framework to coordinate these efforts and link them to policy decision, thus playing a role akin to that of the IPCC for the climate scientific community (Loreau *et al*. 2006).

### (e) Expanding to other issues and research fields

As with many successful new research fields, biodiversity and ecosystem functioning research did not remain confined in its initial scientific boundaries, but expanded its scope to encompass a wide range of fundamental issues in ecology, such as the functioning of food webs (Thébault & Loreau 2006; Duffy *et al*. 2007), the spread of diseases (Keesing *et al*. 2006), the spatial dynamics of metacommunities (Loreau *et al*. 2003), and the relationship between the diversity and stability of ecological systems (McCann 2000; Loreau *et al*. 2002*a*; Ives & Carpenter 2007), where it has provided fresh perspectives.

Progress achieved on the diversity–stability relationship has been particularly significant given the long, controversial history of this issue in ecology. The traditional view that permeated ecology in its early days held that complex, diverse natural ecosystems are inherently more stable than simple or artificially simplified systems (Odum 1953; MacArthur 1955; Elton 1958). Theoretical work by Levins (1970); Gardner & Ashby (1970); May (1972, 1973) and others challenged this traditional view in the early 1970s, and eventually led to an almost diametrically opposite paradigm, i.e. complexity and diversity beget instability, not stability. Although the recent paradigm had rigorous mathematical underpinnings, it also had a number of limitations. In particular, ‘stability’ is really a meta-concept that covers a wide range of different properties. Furthermore, each of these stability properties can be applied to a number of variables of interest at different hierarchical levels, such as individual species abundance, community species composition and aggregate ecosystem properties. The relationship between diversity and stability may be different for different properties and variables (Pimm 1984; Loreau *et al*. 2002*a*; Ives & Carpenter 2007). The recent paradigm specifically concerned the qualitative stability and resilience of communities as ensembles of populations, not the stability of aggregate ecosystem properties. A second important limitation of this paradigm is that it was based on the formalism of autonomous deterministic dynamical systems, which does not consider changes in the external environment and the response of biological systems to these changes through a variety of mechanisms on different time-scales, such as phenotypic plasticity, population dynamics, species replacement and evolution.

**Figure 3.**
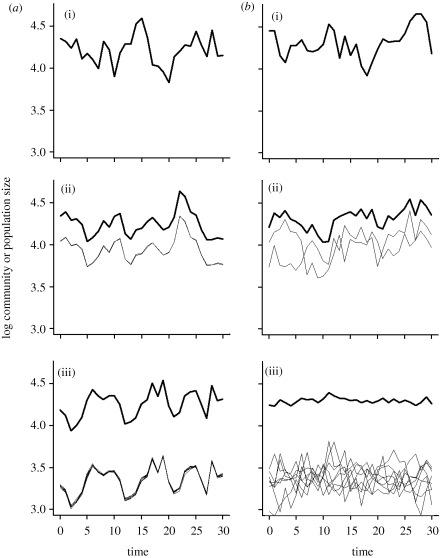
The general mechanism that generates the stabilizing effect of diversity on aggregate community or ecosystem properties. (*b*) When species have asynchronous responses to environmental fluctuations (species have independent environmental responses), their population sizes (thin lines) also fluctuate asynchronously, which reduces the variability of community size (the sum of population sizes, thick lines). Increasing the number of species generally increases the potential for species asynchrony, and hence the stabilization of community properties. (*a*) When species have perfectly synchronous environmental responses, however, increasing the number of species does not contribute to stabilize community size. Modified from Loreau (in press). (*a*,*b*) (i), one species; (*a*,*b*) (ii), two species; (*a*,*b*) (iii), eight species.

Two decisive features distinguish the new theoretical approaches to the diversity–stability relationship developed within the biodiversity and ecosystem functioning research agenda from earlier ones: first, these new approaches explicitly differentiate, and link, stability properties at the population level and at the aggregate community or ecosystem level, and second, they abandon the implicit assumptions that the environment is constant and that populations and ecosystems reach an equilibrium, to explicitly incorporate population dynamical responses to environmental fluctuations. They have led to two closely related hypotheses known as the ‘portfolio effect’ (Doak *et al*. 1998; Tilman 1999) and the ‘insurance hypothesis’ (Yachi & Loreau 1999), which predict a stabilizing effect of species diversity on aggregate ecosystem properties despite or, more exactly, through fluctuations of component species. The general mechanism that makes ecosystem properties less variable in more diverse communities is asynchrony of species' responses to environmental fluctuations driven by niche differences between species (Yachi & Loreau 1999). This asynchrony ensures that as one species decreases sharply in abundance, biomass or productivity, another species decreases less sharply, or even increases, thus compensating partly or wholly for the decrease of the first species. The more species there are and the more asynchronous their environmental responses, the larger the potential for stabilization of aggregate community or ecosystem properties (figure 3). The realization of this potential, however, requires that population dynamics are not strongly destabilized by species interactions at high diversity (Ives & Hughes 2002).

These conclusions extend to complex food webs under some conditions; the outcome in food webs, however, depends critically on how interaction strength varies with species diversity (Thébault & Loreau 2005). Interestingly, both MacArthur's (1955) hypothesis that prey diversity buffers generalist predators against asynchronous fluctuations of their prey and May's (1972, 1973) hypothesis that species diversity generates larger population fluctuations, which have been traditionally opposed, can be obtained with the same model, sometimes even under the same conditions (Thébault & Loreau 2005). This emphasizes that the diversity–stability relationship is a complex, multifaceted one, which does not lend itself to sweeping statements.

Although rigorous empirical support for the new diversity–stability theory is scantier than for the effects of diversity on biomass and production, a few experiments that have manipulated species diversity have provided clear evidence for its stabilizing effect on ecosystem properties in both plant communities (Tilman *et al*. 2006) and aquatic food webs (Steiner *et al*. 2005). Perhaps more surprisingly, recent studies have shown variable responses of population-level stability to species diversity, ranging from negative (Gonzalez & Descamps-Julien 2004; Tilman *et al*. 2006) to positive (Romanuk & Kolasa 2004; Steiner *et al*. 2005). This suggests that current theory may still be missing some significant elements.

Because its initial impetus was provided by the societal relevance of the issues it was addressing, biodiversity and ecosystem functioning research also impacted on social sciences and environmental management. The results of this research supported the work of the Millennium Ecosystem Assessment (2005) on the links between biodiversity, ecosystem services and sustainable development. The value of biodiversity as insurance against the uncertain provision of ecosystem services is being incorporated formally in ecological economics (Armsworth & Roughgarden 2003; Baumgärtner 2007).

### (f) Conclusion

In many ways, the new biodiversity and ecosystem functioning research field represents what I see as a model for the future development of ecology. By addressing a new question of high relevance for both science and society, by challenging existing paradigms, by tightly linking theory and experiments, by building scientific consensus beyond differences in opinion, by integrating fragmented disciplines and research fields, and by connecting itself to other disciplines and management issues, it has contributed to transform ecology not only in content, but also in form.

## 3. Creating an evolutionary ecosystem ecology that integrates community dynamics

I see biodiversity and ecosystem functioning research as it has developed during the past 15 years as the starting point, not the endpoint, of a process of integration of community ecology and ecosystem ecology. This integration still has a long way to go until we can fully understand biodiversity, ecosystem functioning and their multiple linkages. The main obstacle to unification of the two disciplines has been the gap between the macroscopic, holistic perspective of ecosystem ecology and the more microscopic, mechanistic perspective of community ecology. It would be unreasonable to expect this gap to be filled all at once.

One unfortunate consequence of this gap has been the historical divorce between ecosystem ecology and evolutionary biology. The modern theory of evolution sees evolution as a two-step process of mutation and selection that changes gene frequencies within populations. Since genes are carried, expressed and transmitted by individual organisms, the individual organism occupies a central place in this theory, although multilevel selection theory recognizes the potential for higher levels of selection as well (Wilson 1980; Sober & Wilson 1997). As a discipline that studies the overall functioning of ecosystems, ecosystem ecology has had a natural tendency to view ecosystems as integrated units on their own, and hence to search for laws and principles that govern the development and evolution of these higher level units (Fath *et al*. 2001). Although this search has revealed some robust and intriguing patterns, its main weakness lies in the fact that many of the hypotheses proposed to explain these patterns are not explicitly connected to the evolutionary dynamics that takes place at the individual level. As a result, evolutionary biologists have usually disregarded them as wishful thinking. The interplay between the ecological and evolutionary dynamics of communities and ecosystems has received increasing attention recently (Fussmann *et al*. 2007). A major progress would be the emergence of a genuine evolutionary ecosystem ecology that connects aggregate properties at the ecosystem level and evolution of species traits at the individual level (Loreau in press). This endeavour would automatically include community dynamics as a component of this connection.

### (a) The surge of community evolution models

Community genetics is a promising new approach that is providing empirical evidence for predictable effects of heritable traits in a single dominant species on community structure and ecosystem processes (Whitham *et al*. 2006). Community genetics, however, does not consider the reciprocal effects of community structure and ecosystem processes on evolution of these traits, and hence is not yet equipped to tackle evolution of entire ecosystems. But the recent development of community evolution models contains the promise of a genuine theory of ecosystem evolution. These models have so far mainly explored the evolutionary emergence of complex food webs and the constraints generated by this evolutionary history on existing food webs (McKane 2004; Loeuille & Loreau in press). In these models, each species is represented by a set of traits that determine its population dynamics and trophic interactions with other species. These traits are subject to mutations, which create the potential for speciation events. Species may also go extinct as a result of species interactions.

There has been a recent surge of such models, many of which have been developed simultaneously and independently (Caldarelli *et al*. 1998; Christensen *et al*. 2002; Loeuille & Loreau 2005; Ito & Ikegami 2006; Rossberg *et al*. 2006; Bell 2007). The main difference between these models lies in the number and identity of traits they consider. Most of them involve a large number of arbitrary traits (Caldarelli *et al*. 1998; Christensen *et al*. 2002; Loeuille & Loreau 2005; Ito & Ikegami 2006; Rossberg *et al*. 2006; Bell 2007). This makes them flexible, but also relatively abstract and difficult to test empirically. Ito & Ikegami (2006) built a model that includes only two traits for each species, one that describes it as a prey, and the other that describes it as a predator. Loeuille & Loreau (2005) built an even simpler model in which a single evolving trait, body size, determines each species' population dynamics and interactions. The advantages of the latter approach are that it clearly identifies a measurable trait and the ecological trade-offs it generates, and as a result, it makes empirically testable predictions. Body size is well known to play a key role in the physiological and ecological characteristics of species (Kleiber 1961; Peters 1983; Brown *et al*. 2004). Therefore, it is a particularly appropriate trait to consider as a first step towards building a testable theory of food-web evolution.

**Figure 4.**
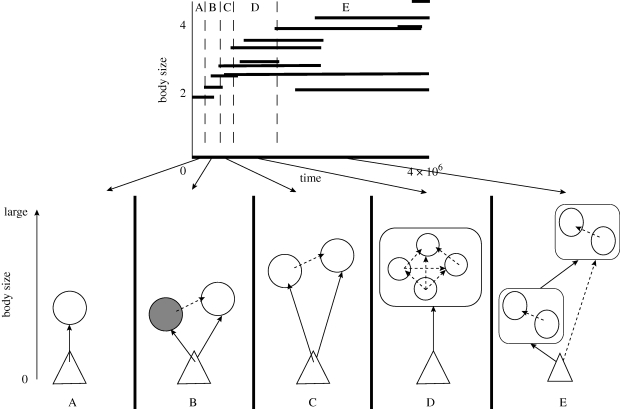
First steps of the evolutionary emergence of a size-structured food web in a community evolution model. The upper panel shows the trait composition of the community through time, while the lower panel details the different steps of the emergence. The simulation starts with a single species that consumes inorganic nutrient (A). Once in a while, mutants appear (here, larger than the resident) and replace their parent (B, in which the grey morph goes to extinction). After several replacements, an evolutionary branching happens, leading to coexistence of the mutant and the resident (C). A rapid diversification then occurs in which several morphs are able to coexist (D). These morphs are then selected to yield differentiated trophic levels (E). Reprinted from Loeuille & Loreau (in press). A, initial condition; B, replacement; C, coexistence, D, diversification; E, divergence.

Our simple model generates complex food webs that emerge by evolution from a single ancestor through a succession of species replacement, coexistence, diversification and divergence processes (figure 4). Diversification is very fast in the beginning, but the food web gradually stabilizes into an evolutionary quasi-equilibrium in which species continue to turn over but food-web structure is relatively stable. These features are found in all existing food-web evolution models. Another intriguing feature of these models is that they are able to generate a wide range of food webs with different structures that strikingly resemble real food webs, from linear food chains with well-defined trophic levels to omnivorous food webs with blurred trophic levels. Even more surprisingly, our simple model is able to fit empirical data on aggregate food-web attributes by varying only two parameters that govern small-scale species interactions, i.e. interference rate and food niche width (Loeuille & Loreau 2005). This is a significant improvement over static food-web models (Cohen *et al*. 1990; Williams & Martinez 2000; Cattin *et al*. 2004), which use aggregate attributes, such as species diversity and connectance, to predict other aggregate attributes, such as food chain length and proportion of omnivores, but which cannot account for the former attributes in the first place. It is also an improvement over other food-web evolution models, which use a large number of poorly specified traits. Interference and food niche width are two individual-level traits that are easy to define and measure, which makes the model's predictions potentially testable.

### (b) Strengths and limitations of community evolution models

Current food-web evolution models may be viewed as extensions of earlier community assembly models (Post & Pimm 1983; Taylor 1989). But I believe that several differences make them more promising and more apt to foster integration across community and ecosystem perspectives. The first and most important difference is that they are based on evolving traits that are defined at the individual level. In contrast, community assembly models have often used population-level phenomenological descriptions of life-history parameters and species interactions such as those found in classical Lotka–Volterra models. The advantage of the former approach is that population, community and ecosystem processes are all emergent properties of individual-level processes and thus are intimately linked through their common dependence on individual-level traits such as body size. Another important difference is that food-web evolution models let species and species interactions emerge spontaneously from the evolutionary dynamics of the system. Community assembly models are more constrained because the range of possible species and food-web configurations is defined *a priori* through the choice of the species pool from which the community is assembled.

Because they use a common set of rules to describe all trophic interactions, existing food-web evolution models are best suited to represent the animal consumer part of natural food webs. They do not account for the basic role division between autotrophs, heterotrophic consumers and heterotrophic decomposers that lies at the core of all ecosystems. Recent theoretical studies have shown that material cycling between autotrophs and heterotrophs can strongly affect the evolution of these functional groups, and, reciprocally, this evolution can strongly affect ecosystem processes (Loreau 1998; de Mazancourt & Loreau 2000; de Mazancourt *et al*. 2001). A few models have explored the evolutionary emergence of nutrient cycling in microbial systems (Downing & Zvirinsky 1999; Downing 2002; Williams & Lenton 2007*b*). These models typically lead to the emergence of a diversity of biochemical guilds with complementary nutrient uptake and release patterns, which collectively recycle nutrients, sustain high biomass and regulate their abiotic environment.

Existing food-web evolution models also lack explicit description of the physical and chemical environment that both affects and is affected by species within ecosystems. The ecosystem engineer and niche construction concepts seek to capture the organisms' power to transform their abiotic environment, which has been too often neglected in ecology and evolutionary biology (Jones *et al*. 1994; Odling-Smee *et al*. 2003). Niche construction generates an adaptive feedback between organisms and their environment, which can yield adaptive regulation of the abiotic environment (Kylafis & Loreau 2008). The interplay between organisms and their physico-chemical environment plays a key role in both evolution and ecosystem functioning. Therefore, an evolutionary ecosystem ecology cannot ignore it.

Although community evolution models are still rudimentary and exploratory, they have the potential to transform ecosystem ecology into a more dynamic discipline that incorporates the perspective of community ecology. Ecosystem evolution models would naturally bridge the gap between the holistic perspective of ecosystem ecology and the mechanistic perspective of community ecology, because both species interactions and ecosystem properties would emerge spontaneously from the interplay between individual-level traits and collective ecosystem-level constraints.

### (c) Moving toward a genuine evolutionary ecosystem ecology

To realize their potential and contribute to create a genuine evolutionary ecosystem ecology, however, these models must evolve beyond their current stage of essentially abstract theoretical explorations. We now need more realistic ecosystem evolution models to generate testable predictions, as well as empirical and experimental data to test these predictions. As I mentioned above, some of the existing food-web evolution models are already able to reproduce aggregate food web patterns accurately. This is encouraging but hardly sufficient to make these models truly predictive tools because it is currently impossible to assess whether they fit empirical data for good reason. There are at least two ways in which their predictive power can be enhanced and tested. First, by collecting simultaneous empirical data on aggregate food-web properties and individual-level traits. This would test the mechanistic basis of the aggregate food-web properties predicted by the models. Second, by developing more mechanistic ecosystem evolution models that take into account the main physical, chemical and biological factors that affect, or are affected by, individuals and the ecosystems they constitute. Such models would provide distinct predictions for different types of ecosystems (e.g. terrestrial versus pelagic), which could then be compared with empirical data from these ecosystems. Experiments using fast-evolving microbial systems could also be used to test some of the basic predictions of these models under relatively simple conditions.

Once appropriately tested against empirical data, ecosystem evolution models have huge application potential. These models could be used to resolve some of the oldest and most controversial issues in ecology and evolutionary biology, such as the respective roles of individual-level and ecosystem-level selection in shaping ecosystem properties (Williams & Lenton 2007*a*). They could also be used to predict both the short-term ecological responses and the long-term evolutionary responses of natural and managed ecosystems to human interventions and anthropogenic disturbances. Their contribution to predicting the long-term consequences of human actions on biodiversity and the long-term future provision of ecosystem services would be particularly valuable.

## 4. Conclusion

In a world that is being transformed by humans at an ever-increasing scale and speed, ecology is bound to gain importance during this century. But in order to help human societies to face growing environmental challenges, ecology will have to deliver relevant scientific knowledge on how ecosystems function and change, how they are linked to human well-being and how humankind can use and transform them in a sustainable way. This requires that ecology transform itself into a more integrated, predictive science. The traditional slicing of ecology into autecology, population ecology, community ecology and ecosystem ecology has some value because different organizational levels obey partly different sets of constraints, and hence partly different laws. But it also has strong limitations because it tends to perpetuate arbitrary divisions and hamper the emergence of unifying perspectives. Current environmental changes affect species, communities and ecosystems indiscriminately. Therefore, a unifying ecological theory is needed to address the consequences of these changes. Recent research on biodiversity and ecosystem functioning has contributed to this goal in several different ways. Creating a genuine evolutionary ecosystem ecology that links the evolution of species traits at the individual level, the dynamics of species interactions, and the overall functioning of ecosystems would give new impetus to this much-needed process of unification across ecological disciplines.
